# Exposure to Apoptotic Activated CD4^+^ T Cells Induces Maturation and APOBEC3G- Mediated Inhibition of HIV-1 Infection in Dendritic Cells

**DOI:** 10.1371/journal.pone.0021171

**Published:** 2011-06-16

**Authors:** Venkatramanan Mohanram, Ulrika Johansson, Annette E. Sköld, Joshua Fink, Sushil Kumar Pathak, Barbro Mäkitalo, Lilian Walther-Jallow, Anna-Lena Spetz

**Affiliations:** Center for Infectious Medicine, Department of Medicine, Karolinska Institutet, Karolinska University Hospital Huddinge, Stockholm, Sweden; University Hospital Zurich, Switzerland

## Abstract

Dendritic cells (DCs) are activated by signaling via pathogen-specific receptors or exposure to inflammatory mediators. Here we show that co-culturing DCs with apoptotic HIV-infected activated CD4^+^ T cells (ApoInf) or apoptotic uninfected activated CD4^+^ T cells (ApoAct) induced expression of co-stimulatory molecules and cytokine release. In addition, we measured a reduced HIV infection rate in DCs after co-culture with ApoAct. A prerequisite for reduced HIV infection in DCs was activation of CD4^+^ T cells before apoptosis induction. DCs exposed to ApoAct or ApoInf secreted MIP-1α, MIP-1β, MCP-1, and TNF-α; this effect was retained in the presence of exogenous HIV. The ApoAct-mediated induction of co-stimulatory CD86 molecules and reduction of HIV infection in DCs were partially abrogated after blocking TNF-α using monoclonal antibodies. APOBEC3G expression in DCs was increased in co-cultures of DCs and ApoAct but not by apoptotic resting CD4^+^ T cells (ApoRest). Silencing of APOBEC3G in DC abrogated the HIV inhibitory effect mediated by ApoAct. Sequence analyses of an *env* region revealed significant induction of G-to-A hypermutations in the context of GG or GA dinucleotides in DNA isolated from DCs exposed to HIV and ApoAct. Thus, ApoAct-mediated DC maturation resulted in induction of APOBEC3G that was important for inhibition of HIV-infection in DCs. These findings underscore the complexity of differential DC responses evoked upon interaction with resting as compared with activated dying cells during HIV infection.

## Introduction

DCs are professional antigen-presenting cells that play a central role in connecting innate and adaptive immunity[1]. DCs residing in peripheral tissues engulf both foreign microbial material and dying cells of the host. Maturation signals are, however, required for DCs to undergo phenotypic and functional changes to acquire a fully competent antigen-presenting capacity. Maturation of DCs is a process that includes a transient increased capacity for antigen uptake; migration to draining lymph nodes; and simultaneous upregulation of molecules including chemokine receptors, major histocompatibility complex (MHC) class I and II molecules, and co-stimulatory molecules [1], [2]. DC maturation is triggered by CD40 ligand, danger associated molecular pattern released from certain dying cells and by pro-inflammatory cytokines, such as tumor necrosis factor-α (TNF-α), IL-1β, IL-6, and interferon-α (IFN-α) [3]. In addition, infected apoptotic cells may provide a DC maturation signal through interactions with pattern recognition receptors including members of the Toll-like receptor (TLR) family [4]–[6]. DCs are located in mucosal and lymphoid tissues and because of their localization in the mucosa, are likely to be among the first cells to encounter invading HIV particles [7]. It has been shown that DCs can be infected by HIV and support viral replication, suggesting that HIV-infected DCs may be a viral reservoir *in vivo* [8]. Experiments using human cervical explants models showed that emigrating DCs had captured HIV and were capable of transmitting the virus to CD4^+^ T cells in *trans* [9]. In vitro experiments further support a role for DCs in transmitting HIV to CD4^+^ T cells which was demonstrated to occur both in *cis* and *trans* [7].

DC maturation suppresses HIV infection through multifaceted mechanisms that involve decreased viral fusion [10], a block of reverse transcription [11] and a post-integration restriction that has been proposed to exist at the transcriptional level [3]. There are several described intrinsic factors that act at discrete steps in the viral life cycle to mediate suppression of HIV replication in DCs. These include chemokines and their receptors, which play a central role in HIV infection and disease progression. HIV uses the chemokine receptors CCR5 and CXCR4 as the main co-receptors for viral entry [12]. The chemokines MIP-1α, MIP-1β, and RANTES, which bind to CCR5, have potent anti-HIV activity [13]. Although many cytokines were initially described as having anti-HIV activities, subsequent or simultaneous studies using different experimental conditions have shown that they may also activate virus production [14], [15]. TNF-α was identified as the predominant inducer of HIV production in supernatants from both monocyte and lymphocyte cultures [16], [17]. The proviral activity is dependent on the activation of NF-κB present in the cytoplasm of both monocytes and lymphocytes [18]–[20]. The dual activity of TNF-α with regard to HIV replication was later suggested by a study showing inhibition of HIV replication before proviral DNA integration [21]. TNF-α was furthermore shown to induce DC maturation, leading to down-regulation of HIV co-receptors [22]. Hence, there are several cytokines, including TNF-α, IFN-γ, TGF-β and MCP-1, that have either pro-HIV or anti-HIV effects depending on the experimental system and/or the types of cells analyzed [14], [15]. In addition type I interferons were shown to induce the restriction factor APOBEC3G [23]. Members of the APOBEC family of proteins confer intrinsic immunity to retroviral infection [24]. APOBEC3G levels are increased upon DC maturation, suggesting a role for APOBEC3G as a possible candidate for viral restriction in maturing DCs exposed to HIV [23], [25]. APOBEC3G is restricting HIV replication by deamination of viral cDNA cytidine during reverse transcription. This will lead to a characteristic induction of G-to-A hypermutations. However, some studies suggest deaminase-independent effects by APOBEC3G proteins interfering with steps in reverse transcription or integration (reviewed in [23]).

Uptake by DCs of apoptotic bodies originating from HIV-infected cells *in vitro* results in activation of CD4^+^ and CD8^+^ T cells [26]–[28]. Antigens contained in apoptotic bodies are introduced into MHC class I presentation pathways for cross-presentation and stimulation of HIV-specific CD8^+^ T cells. We recently demonstrated that activated, but not resting, apoptotic peripheral blood mononuclear cells (PBMCs) are able to induce maturation of DCs in terms of up-regulation of costimulatory molecules (CD80 and CD86) and induction of pro-inflammatory cytokine release [29]. In the present study, we determined whether apoptotic CD4^+^ T cells support DC maturation and whether HIV infection interferes with this process. We show that apoptotic HIV-infected (ApoInf) and uninfected activated CD4^+^ T cells (ApoAct) provide maturation signals to DCs that result in the release of TNF-α, MIP-1α, MIP-1β and MCP-1. Furthermore, upregulation of CD86 molecules in DCs was reduced in the presence of anti-TNF-α. We further found a significantly reduced percentage of HIV-infected DCs when they were co-cultured with ApoAct as compared with DCs co-cultured with apoptotic resting CD4^+^ T cells (ApoRest); this effect was partly blocked in the presence of anti-TNF-αmAbs. We detected upregulation of APOBEC3G mRNA expression in DCs co-cultured with ApoAct but not with ApoRest. In addition, we demonstrated induction of G-to-A hypermutations in *env*-DNA isolated from HIV infected DCs co-cultured with ApoAct but not with ApoRest, as a sign of APOBEC3 activity in mature monocyte derived DCs These findings may have relevance for cell-associated HIV transmission and pathogenesis, as dying uninfected and HIV-infected cells constitute a characteristic hall mark in acute and chronic HIV infection [30], [31].

## Materials and Methods

### Human subjects and blood collection

Buffy coats from healthy human blood donors were obtained from the blood bank at Karolinska University Hospital Huddinge. Ethical approval was obtained from the medical ethics committee in Stockholm.

### *In vitro* differentiation of DCs

CD14^+^ monocytes were enriched from buffy coats by negative selection using RosetteSep Human Monocyte Enrichment (1 ml/10 ml blood; Stem Cell Technologies, Vancouver, BC, Canada). Monocytes were separated using Lymphoprep density gradient (Nycomed, Oslo, Norway) and were cultured for 6 days in DC medium containing RPMI 1640 (GIBCO Life Technologies, Paisley, United Kingdom), supplemented with 1% HEPES (*N*-(2-hydroxyethyl)piperazine-*N*'-2-ethanesulfonic acid) (GIBCO), 2 mM l-glutamine (GIBCO), 1% streptomycin (GIBCO), and 1% penicillin (GIBCO), 10% endotoxin-free fetal bovine serum (FBS) (GIBCO), recombinant human cytokines IL-4 (6.5 ng/ml; R&D Systems, Minneapolis, MN) and granulocyte-macrophage colony-stimulating factor (GM-CSF; 250 ng/ml; Peprotech; London, United Kingdom) to obtain immature DCs [32].

### Purification and activation of CD4^+^ T cells

CD4^+^ T cells were enriched from buffy coats obtained from whole blood by negative selection using RosetteSep Human CD4^+^ T Cell Enrichment (1 ml/10 ml blood; Stem Cell Technologies). CD4^+^ T cells were separated using Lymphoprep density gradient (Nycomed). Cells were frozen in FBS and 10% dimethylsulfoxide (DMSO) or were added to flasks containing monoclonal anti-human CD3 (10 µg/ml; clone OKT 3; Ortho Biotech Inc., Raritan, NJ) that had been adhered to the plastic overnight at 4°C and soluble monoclonal anti-human CD28 (2 µg/ml; L293; BD Biosciences, San Diego, CA). After 24 hours of stimulation, cells were frozen in FBS/DMSO. On the day of experiment, frozen T cells were thawed, washed, and induced to undergo apoptosis by γ-irradiation (150 Gy) as described [29], [33]-[36].

### HIV growth and preparation

The HIV isolates HIV_BaL_ (which uses CCR5 receptors) and HIV_IIIB_ (which uses CXCR4 receptors) were obtained from the National Institutes of Health (NIH) AIDS Research and Reference Reagent Program, Division of AIDS, National Institute of Allergy and Infectious Diseases (NIAID, NIH, Bethesda, MD). HIV isolates were grown in PBMC cultures stimulated with phytohemagglutinin (PHA; 2.5 µg/ml; Sigma, St Louis, MO) and IL-2 (75 IU/ml; Chiron, Emeryville, CA). Primary virus was isolated from HIV-infected patient blood by co-culture with PHA-activated donor PBMCs. To concentrate the virus and to minimize the presence of bystander activation factors in the supernatant that could induce DC maturation, virus stocks were ultracentrifuged (138,000×*g* for 30 minutes at 4°C. (Beckman L-80 Ultra-centrifuge, rotor 70.1; Beckman Coulter, Fullerton, CA), and the virus pellets were resuspended in RPMI 1640 with 10% FBS to obtain a virus concentrate. An aliquot of each viral stock was thawed and titrated for infectivity using a limiting dilution culture method with PHA-activated PBMCs (mix of 3 different donors) [37]. The 50% tissue culture infectious dose (TCID50) was calculated by the method of Reed and Muench as described in [38]. The titers of virus stocks were 1.7×10^6^/ml TCID)_50_ for both HIV_BaL_ and HIV_IIIB_. The primary isolates (207 and 208) had a TCID_50_ of 1.0×10^3^/ml and 4.0×10^4^/ml, respectively.

### HIV infection of CD4^+^ T cells and DCs

Activated (24 hours with CD3 and CD28 monoclonal antibodies (mAbs) as described above) CD4^+^ T cells were incubated with HIV_BaL_ (3000–6000 TCID_50_) in the presence of IL-2. The percentage of infected cells was analyzed by intracellular staining for the HIV Gag protein p24 at 3, 4, 5, 6, 7, and 10 days after infection [32], [33]. The highest percentage of cells infected was detected at days 4, 5, and 6; thereafter infection percentages declined (data not shown). Batches of infected cells collected at day 3–4 were frozen in FBS/DMSO until use. For infection of DCs, 3000–6000 TCID_50_ of HIV_BaL_ and HIV_IIIB_, and 500 TCID_50_ of the primary isolates were used. The percentage of infected DCs was determined by intracellular p24 staining after 7 days of infection.

### Quantification of HIV protein in T cells and DCs

The percentage of HIV_BaL_-infected DCs and CD4^+^ T cells was determined by intracellular staining for p24 [32], [33]. Cells were first stained for cell surface markers and then fixed in 2% formaldehyde (Sigma) for 10 minutes at room temperature. Cells were washed in saponin solution consisting of PBS with 2% FBS, 2% HEPES, and 0.1% saponin (Sigma), to allow permeabilization of the cell surface membrane, and then incubated for 1–2 hours at 4°C with a p24 mAb (clone KC57; Coulter, Hialeah, FL) or the corresponding isotype control antibody. Intracellular p24 expression was assessed by a FACSCalibur flow cytometer (Becton Dickinson, San Jose, CA) and data were analyzed using CellQuest Software (Becton Dickinson, San Jose, CA).

### DC and apoptotic CD4^+^ T cell co-cultures

Differentiated, immature DCs were co-cultured with irradiated CD4^+^ T cells at a ratio of 1∶2 DC:T cells in DC medium. Conditioned medium (CM) was prepared by co-culturing DCs with ApoAct for 24 hours. In some experiments, CM was added to the DC cultures at 40% of the total volume. Supernatants were collected from co-cultures at 4, 8, and 24 hours. The TLR4 agonist LPS (100 ng/ml; Sigma) was used as a positive control for activation and maturation of DCs. DCs were exposed to HIV_BaL_, and apoptotic cells or CM was added at 0, 2, or 16 hours. For cytokine blocking experiments, CM was pre-incubated for 2 hours at 37°C with blocking antibodies alone or in combination (all obtained from R&D Systems): anti-MIP-1α (clone 14215; 2.5 mg/ml), anti-MIP-1β (clone 24006; 1 mg/ml), anti-IFN-γ (clone 25718; 4 mg/ml), anti-RANTES (clone 21445; 2.5 mg/ml), and anti-TNF-α (clone 1825; 0.5 mg/ml). In DC/ApoAct co-cultures, anti-TNF-α was added at the beginning of the co-culture. DCs were analyzed by flow cytometry for p24 and CD86 expression 7 days after infection.

### Phenotypic characterization of DCs and T cells

DCs were stained with the following anti-human mAbs: CD1a (clone NA1/34; DAKO, Glostrup, Denmark), CD14 (clone TÜK4; DAKO), CD3 (clone SK7; BD Biosciences), CD83 (clone HB15e; BD Biosciences), and CD86 (clone 2331/FUN-1; BD Biosciences). T cells were stained with anti-human mAbs CD19 (clone HD37; DAKO), CD3 (clone SK7; BD Biosciences), CD4 (clone RPA-T4; BD Biosciences), CD8 (clone SK-1; BD Biosciences), CD25 (clone 2A3; BD Biosciences), and CD69 (FN50; BD Biosciences). Cell surface expression was measured by a FACSCalibur flow cytometer (Becton Dickinson). Co-culture samples were collected at 72 hours or 7 days, washed, and incubated with the previously mentioned CD1a, CD83, CD86, and p24 mAbs, as indicated.

### Cytokine and chemokine production

Supernatants from apoptotic CD4^+^ T cells and DC co-cultures were analyzed for cytokines and chemokines with the Bio-Plex assay (Biosource, Nivelles, Belgium). A Luminex reader (Luminex Corporation, Austin, TX) was then used to simultaneously quantify the concentration of IL-2, RANTES, IL-10, IL-12, TNF-α, MCP-1, MIP-1α, and MIP-1β in the supernatants.

### Quantification of HIV-long terminal repeat (LTR) in DCs

Genomic DNA was extracted from infected DCs using the QIAamp DNA Mini Kit (Qiagen Inc., Valencia, CA). HIV-LTR sense and anti-sense primer sequences were 5′-GCCTCAATAAAGCTTGCCTTGA-3′ and 5′-GGGCGCCACTGCTAGAGA-3′. The probe sequence was 5′-6-FAM-CCAGAGTCACACAACAGACGGGCACA-TAMRA-3′, and the resulting amplicon was 121 bp. Primer and probe design was based on published sequences [39] and were from Applied Biosystems, Foster City, CA. Real-time PCR reactions were performed in 10-µl reactions (Applied Biosystems) using the 7500 Real-Time PCR System (Applied Biosystems). For absolute quantification of HIV-LTR, a control plasmid was constructed by cloning the 121-bp HIV-LTR amplicon into pCR®4-TOPO® (Invitrogen Life Science). Plasmid copy number was quantified as described [40].

### HIV-1-integration Alu-PCR assay

Total cell DNA was isolated at 72 h after infection with a QIAamp blood isolation kit (Qiagen). Integrated HIV-1 DNA was measured by two-step Alu-PCR [6]. In the first round of pre-amplification PCR, Alu-LTR sequences were amplified with an HIV-1-specific primer (LTR R region) in combination with a primer that anneals to the abundant genomic Alu repeats. The HIV-1-specific primer was extended with a marker region at the 5′ end, which was used for specificity in the second-round PCR. The second round was nested quantitative real-time PCR of the first-round PCR products with primers annealing to the aforementioned marker region in combination with a HIV-1-specific primer (LTR U5 region). Primer sequences were as follows: first round, HIV-1 LTR R forward, 5′-ATGCCACGTAAGCGAAACTGCTGGCTAACTAGGGAACCCACTG-3′ (marker sequence underlined); Alu reverse, 5′-TCCCAGCTACTGGGGAGGCTGAGG-3′; second-round marker forward, 5′-ATGCCACGTAAGCGAAACTG-3′; HIV-1 LTR U5 reverse, 5′-CACACTGACTAAAAGGGTCTGAGG-3′. Samples were assayed at two concentrations to ensure that PCR inhibitors were absent. Dilutions were prepared with genomic DNA from uninfected cells to ensure that the number of Alu sites per reaction mixture remained constant. For monitoring of the signal contributed by non-integrated HIV-1 DNA, the first-round PCR was also done with the HIV-1-specific primer alone as a control. HIV-1 integration was normalized relative to GAPDH DNA. For relative HIV-1 integration, the value obtained for BaL-infected DCwere set to 1 for each donor and compared with DC from the same donor co-cultured with ApoAct or ApoRest.

### Real-Time PCR analysis and silencing of APOBEC3G

RNA was extracted either from freshly isolated DCs or from DCs co-cultured with LPS or with ApoRest or ApoAct as described above. RNA was isolated using the RNeasy Mini Kit (Qiagen). RNA was reverse transcribed using High Capacity cDNA Reverse Transcription Kit (Applied Biosystems). Amplification of APOBEC3G, 18s RNA and GAPDH cDNA was performed using the 7500 Real-Time PCR System (Applied Biosystems) and 6-carboxyfluorescein dye-labeled TaqMan MGB probes and primers (Applied Biosystems) [41]-[43]. We did a homology BLAST for the primer-probe, which is highly specific to APOBEC3G. The reference sequence ID is NM_021822.3, NCBI chromosome location is Chr. 22 - 39473010 – 39483748. The primer- probe was designed to target 5- 6 exon boundary of APOBEC3G and the amplicon length was 80 bp. Cycle threshold values for APOBEC3G were normalized to the value for GAPDH or 18S in control experiments. Data are presented as fold changes in mRNA copy number in the DCs co-cultures as compared to mRNA in DCs cultured in medium only. DCs were transfected with 25 nM siRNA with the transfection reagent DF4 (Dharmacon, Thermo Fisher Scientific) and were used for experiments 36 hours after transfection [6]. The siRNA (SMARTpool; Dharmacon) specific for APOBEC3G was purchased from Dharmacon (M-013072-00). Non-targeting siRNA (D-001206-13; Dharmacon) served a control. This protocol resulted in a transfection efficiency of nearly 100%, as determined by flow cytometry of cells transfected with siGLO RISC-Free Control siRNA (D-001600-01; Dharmacon), and 79–90%of silencing of expression of APOBEC3G was achieved as verified by real-time PCR analysis.

#### HIV-1 *env* sequencing and analysis

Total cell DNA was isolated 72 h after infection with a QIAamp DNA mini kit (Qiagen). HIV-1 *env* V1-V5 gene region was amplified by performing high-fidelity nested PCR using an EasyA kit (Stratagene). The primers used were as follows: Round 1 (product size 1415 bp) Forward 5′ TTGCAATAGAAAAATTCTCCTC 3′, Reverse (5′CCTGGTGGGTGCTACTCCTA 3′). Round 2 (product size 1098 pb) Forward 5′ CCATGTGTAAAGTTAACCCC 3′, Reverse, 5′ ATGAGGGACAATTGAGAAGTGTCTAG 3′, PCR condition as described in [44]. Amplicons were purified by using a high pure PCR product purification kit (Roche), and cloned into the TA cloning vector provided in a TOPO TA cloning kit (Invitrogen) in accordance with the manufacturer's instructions. Plasmids were isolated using Genejet plasmid mini prep kit (Fermentas) and the plasmids were sent to Eurofins MWG Operon, Germany for sequencing. Primers M13 forward (5′ GTAAAACGACGGCCAG 3′) and M13 reverse (5′ CAGGAAACAGCTATGAC 3′) were used for sequencing. G-to-A mutations were analyzed by using the program Hypermut 2.0 that is freely available at http://www.hiv.lanl.gov/content/sequence/HYPERMUT/hypermut.html [45].

## Results

### Up-regulation of co-stimulatory molecules on DCs after co-culture with apoptotic activated CD4^+^ T cells; uninfected (ApoAct) and HIV-infected (ApoInf)

We previously demonstrated that apoptotic anti-CD3/CD28–activated PBMCs, but not apoptotic resting PBMCs, can induce DC maturation [29]. Here we first investigated whether apoptotic HIV-infected activated CD4^+^ T cells (ApoInf) are able to induce DC maturation. Primary CD4^+^ T cells were activated with CD3 and CD28 mAbs for 24 hours before infection with HIV. The efficiency of T cell activation was determined by expression of CD25 and CD69. After activation, the percentage of CD25^+^CD4^+^ T cells was 49–66%, and the percentage of CD69^+^CD4^+^ T cells was 44–48%, as compared with 0.7–13% and 0.4–6.3%, respectively, before activation (data not shown). We next infected activated CD4^+^ T cells with HIV_BaL_. The infection efficiency, as determined by intracellular p24 staining, was 28–43% (data not shown). Batches of cells were frozen, and on the day of experiment, cells were thawed, washed, and induced to undergo apoptosis by γ-irradiation as described [29], [33]–[36]. In the majority of experiments, γ-irradiated T cells were added to the DC cultures immediately after radiation exposure to allow early apoptotic events to occur in the co-cultures.

**Figure 1:**
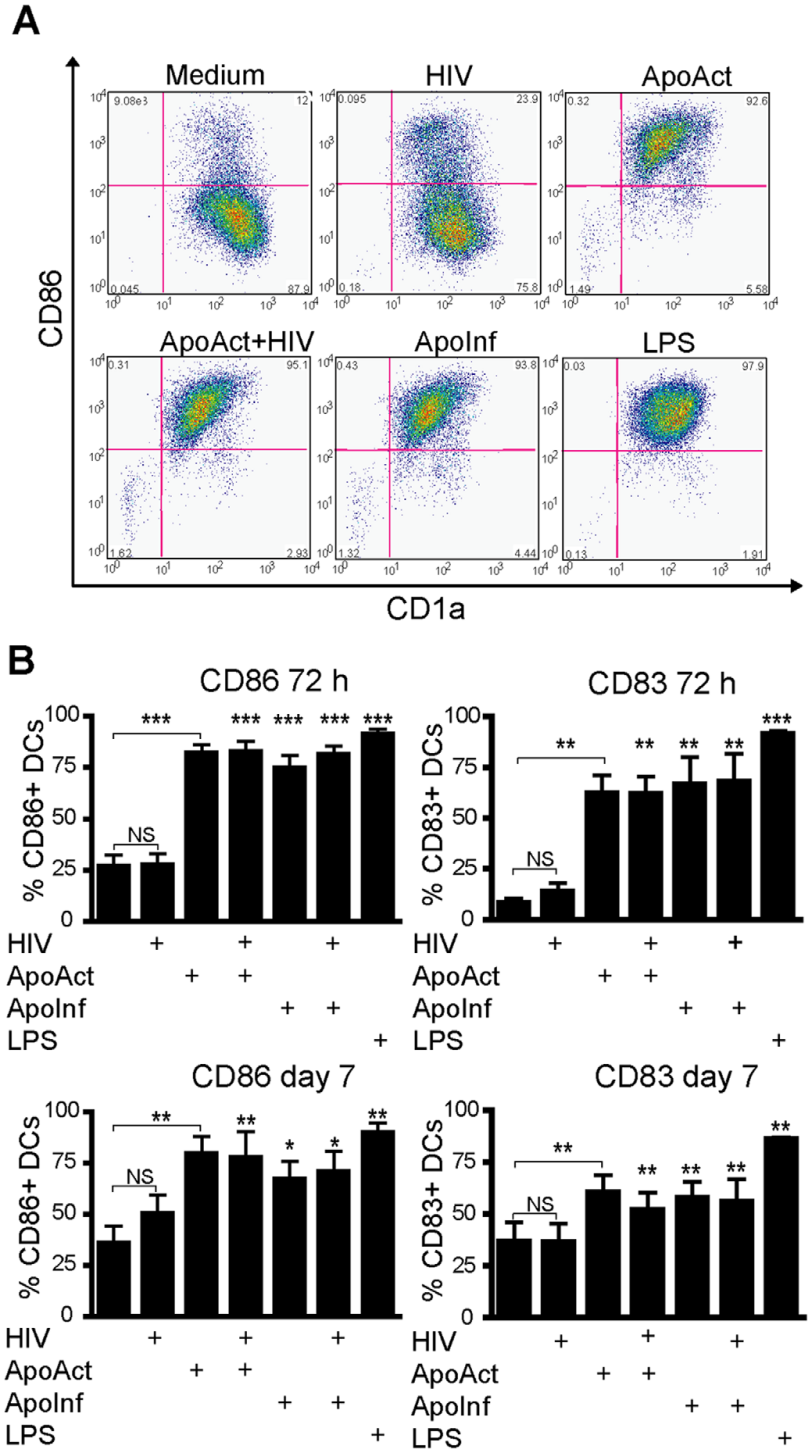
ApoAct or ApoInf induce CD86 and CD83 expression in human DCs. Human *in vitro*–differentiated monocytes cultured for 6 days in the presence of IL-4 and GM-CSF were used as the source of human immature DCs. (A) Immature DCs were co-cultured with apoptotic allogeneic CD4^+^ T cells for 72 hours or 7 days and then analyzed for expression of CD86 molecules by flow cytometry. Gates were set on large CD1a^+^CD3^–^ cells. LPS, which is a potent DC activator, was used as a positive control, and DCs cultured in medium only were used as a negative control. Immature DCs were cultured in medium alone or in the presence of LPS (LPS), HIV_BaL_ (HIV), apoptotic anti-CD3– and anti-CD28–activated CD4^+^ T cells (ApoAct), ApoAct in the presence of exogenous HIV_BaL_ (ApoAct+HIV), or apoptotic HIV_BaL_-infected activated CD4^+^ T cells (ApoInf). Representative flow cytometry data from one donor after 7 days of co-cultures are shown. (B) The average percentages of CD86^+^ DCs ± SD for 11 donors and of CD83^+^ DCs ± SD for 7 donors are depicted. Data from at least four independent experiments are included. Significant differences compared with medium control were assessed by the non-parametric Mann-Whitney *U*-test, and significance is indicated by *p<0.05, **p<0.01, and ***p<0.001.

We used *in vitro*–differentiated monocytes cultured for 6 days in the presence of IL-4 and GM-CSF as the source of human immature DCs, which are defined by the expression of CD1a; absence of CD14; and low expression of CD40, CD80, CD86, and CD83 [29], [32]. These immature DCs were co-cultured with ApoAct or ApoInf for 72 hours or 7 days and then analyzed for expression of the co-stimulatory molecule CD86 (Figure 1). Representative flow cytometric analyses are depicted in Figure 1A, and a summary of 11 donors is shown in Figure 1B. The percentage of CD86^+^ DCs was 91.1±2.5% after LPS stimulation and the background medium control was 26.9±5.3% after 72 hours. DCs exposed to HIV_BaL_ viral particles did not significantly up-regulate CD86 after 72 hours or 7 days of culture as compared with the medium control (Figure 1B) although some donors had a low induction of CD86 (Figure 1A). It should be noted that the virus stock used in the present study was ultra-centrifuged to remove cell debris and possible bystander soluble factors. Co-culture with ApoAct or ApoInf, on the other hand, resulted in a robust and significant induction of CD86 as compared with medium control both after 72 hours (*p<*0.001) and 7 days (*p<*0.01 and *p<*0.05, respectively) of culture (Figure 1B). It is conceivable that the local milieu *in vivo* upon DC encounter with infected or uninfected apoptotic cells will contain viral particles. We therefore investigated whether this mechanism for induction of DC maturation was functional in the presence of viral particles. We found that the maturation signal provided by the ApoAct or ApoInf occurred even in the presence of free HIV_BaL_, and the efficiency of induced CD86 expression was comparable to that of the positive control.

The expression of CD83, another molecule associated with DC maturation and functional antigen-presenting capacity, showed an expression pattern that was similar to that of CD86 but with a tendency of lower expression than CD86. These findings show that both ApoAct and ApoInf are able to provide a maturation signal to immature DCs even in the presence of exogenous HIV.

### Cytokines and chemokines are secreted after co-culture of DCs with ApoAct and ApoInf

**Figure 2:**
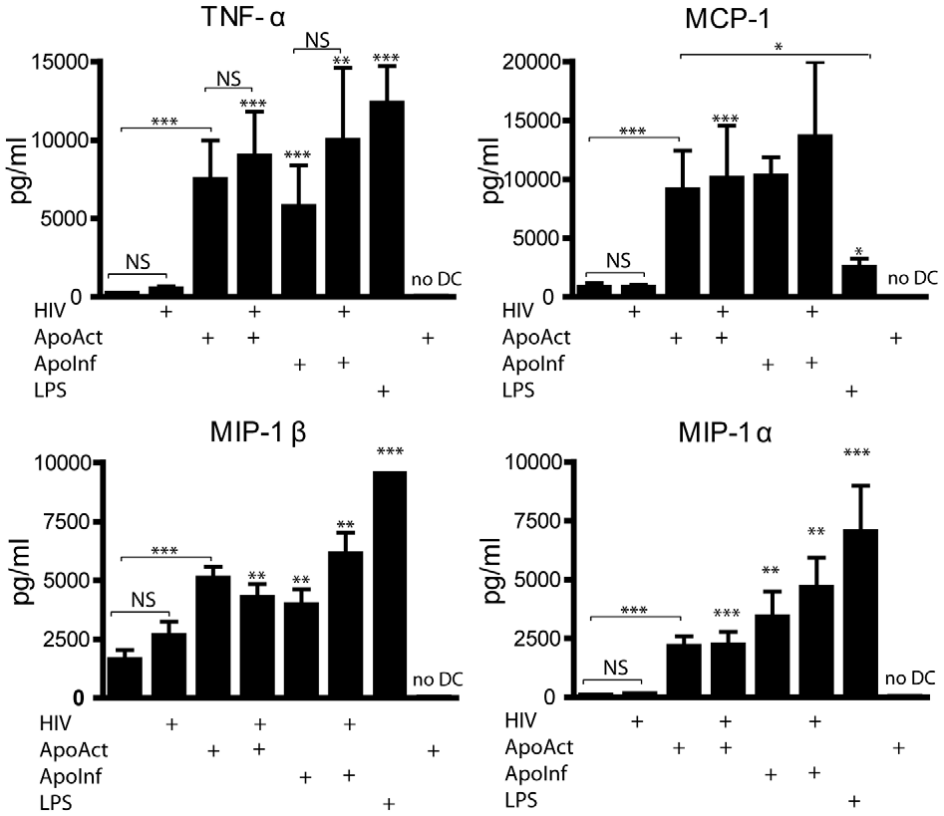
Cytokine and chemokine released from DCs exposed to ApoAct or ApoInf. Immature DCs were co-cultured with activated apoptotic allogeneic CD4^+^ T cells for 24 hours, and culture supernatants were analyzed for the presence of TNF-α, MCP-1, MIP-1α and MIP-1β. DCs were cultured in medium alone (negative control) or in the presence of LPS, which is a potent DC activator (positive control). DCs were exposed to HIV_BaL_ (HIV), anti-CD3– and anti-CD28–activated apoptotic CD4^+^ T cells (ApoAct), ApoAct in the presence of HIV_BaL_ (ApoAct+HIV), apoptotic anti-CD3– and anti-CD28–activated CD4^+^ T cells infected with HIV_BaL_ (ApoInf), or ApoInf in the presence of exogenous HIV_BaL_ (ApoInf+HIV). Control wells included ApoAct without any DCs (no DC). The results shown are the mean ± SD from at least seven donors except for the MCP-1 analysis with ApoInf which shows data from two donors. Significant differences compared with medium control were assessed by non-parametric Mann-Whitney *U*-test and Kruskal-Wallis test with Dunn's multiple comparison test and are indicated by *(*p*<0.05), ** (*p*<0.01) and *** (*p*<0.001), respectively. Non-significant, NS.

To address whether cytokine production was induced in DCs after exposure to ApoAct and/or ApoInf, we collected supernatants from the co-cultures after 4, 8 and 24 hours and used a multiplexed cytokine assay for simultaneous analyses of MIP-1α, MIP-1β, TNF-α, IL-10, IL-12, and MCP-1. There was a significant induction of TNF-α from the co-cultures with apoptotic anti-CD3– and anti-CD28–activated CD4*^+^* T cells either uninfected (*p<*0.001) or HIV-infected cells (*p<*0.001), as compared with medium control after 24 hours of co-culture (Figure 2). TNF-α was detected in co-culture supernatants even when exogenous HIV_BaL_ was present in the cultures and reached quantities in a similar range as LPS stimulation. We did not detect any secretion of the cytokines analyzed from the ApoAct *per se* (denoted as “no DC”; Figure 2). Co-cultures with apoptotic resting CD4^+^ T cells (ApoRest) or apoptotic neutrophils did not result in any secretion of the mentioned cytokines (data not shown and [29]). We could not detect any significant production of IL-10 or IL-12p70 after co-culturing with ApoAct, regardless of infection status (data not shown). Production of the chemokine MCP-1, which enhances mucosal IgA secretion and cytotoxic T cell responses [46], was detected in supernatants from co-cultures of DCs and ApoAct (*p<*0.001 as compared with medium control; Figure 2). MCP-1 production also occurred in the presence of exogenous HIV_BaL_. There were significantly increased quantities of MCP-1 secreted in co-cultures with ApoAct as compared with LPS (*p<*0.05). We also detected significant induction of MIP-1α and MIP-1β expression in co-cultures with ApoAct, regardless of HIV infection (Figure 2). Altogether, these findings suggest that both ApoAct and ApoInf are able to induce pro-inflammatory cytokine and chemokine production in DCs.

### Reduced percentage of HIV-infected DCs detected in co-cultures with ApoAct but not ApoRest

**Figure 3:**
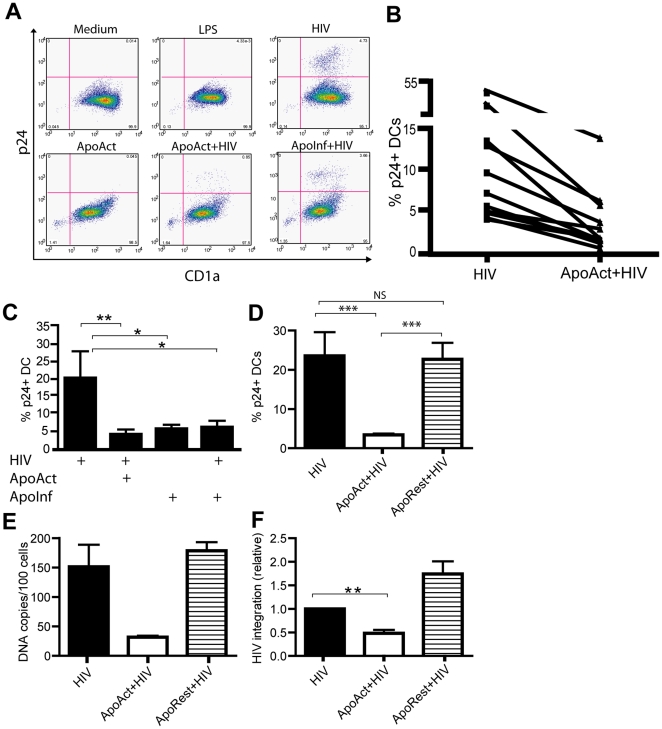
Reduced percentage of HIV-infected DCs detected after co-culturing with ApoAct but not ApoRest. Immature DCs were exposed to HIV_BaL_ (HIV), apoptotic anti-CD3– and anti-CD28–activated allogeneic CD4^+^ T cells (ApoAct), ApoAct in the presence of HIV_BaL_ (ApoAct+HIV), apoptotic anti-CD3– and anti-CD28–activated allogeneic CD4^+^ T cells infected with HIV_BaL_ (ApoInf), or ApoInf in the presence of exogenous virus (ApoInf+HIV). The percentage of infected DCs was assessed by flow cytometry analysis of intracellular p24 staining after 7 days. (A) Representative results from one donor and (B) paired results from eleven donors, both after 7 days of infection, are shown. (C) The average percentage of p24^+^ DCs ± SD after 7 days in eight donors is shown. (D) Data comparing the percentage of p24^+^ DCs in the presence of HIV_BaL_ after co-culturing with ApoAct (ApoAct+HIV) and apoptotic resting CD4^+^ T cells (ApoRest+HIV). Results are shown as the mean ± SD of p24^+^ DCs from additional paired eight donors. Significant differences were assessed by the non-parametric Wilcoxon test and are indicated by *p<0.05, **p<0.01, and ***p<0.001. (E) The number of viral DNA copies/100 cells as assessed by quantitative real-time PCR for three donors is shown. (F) Integration of HIV DNA was assessed by Alu-PCR. Data depict five different donors. Significant differences were assessed by the non-parametric test and are indicated by *p<0.05, **p<0.01.

The finding that several cytokines were released into the superfonatants, including those with anti-HIV activity [13], prompted us to ask whether co-culturing with ApoAct could influence the efficiency of virus infection in DCs. We measured HIV infection by determining the percentage of cells expressing intracellular p24 antigen, as described (Figure 3A) [32], [33]. Addition of 3′-azido-3′-deoxythymidine (AZT) to the DC cultures inhibited detection of p24, which showed that intracellular p24 as detected by flow cytometry was the result of productive infection in DCs [32].

Immature DCs were exposed to HIV_BaL_; there was large variability between donors with regard to HIV infection efficiency, ranging from 4.0 to 46.4% after 7 days (Figure 3B). Co-culture of DCs with ApoAct, either uninfected or HIV-infected, resulted in a significantly reduced percentage of p24^+^ DCs as compared with DCs exposed only to HIV_BaL_ (Figure 3B,C; *p<*0.01 and *p<*0.05, respectively). There was no significant reduction in p24 expression in DCs after exposure to ApoRest (Figure 3D). We detected similar induction of CD86 expression and reduction in percentage of p24 positive DCs using either autologous or allogeneic ApoAct (Figure S1). The critical step to gain the capacity to induce DC maturation and reduction in HIV-infection was the activation step (Figure 3D).We also measured HIV infection efficiency by quantitative PCR. There was an almost tenfold reduction in viral DNA copies in samples co-cultured with ApoAct but not ApoRest (Figure 3E). Alu-PCR was used to determine the relative proportion of integrated HIV DNA to investigate whether the inhibition occurred pre- or post-integration. The values obtained in the HIV infected DC were set to one in all donors. There was a significantly reduced value obtained in DCs co-cultured with ApoAct, suggesting that a block occurred at least in part prior to HIV integration (Figure 3F). We next performed dose-response experiments to investigate the DC/ApoAct concentration required to obtain a reduced percentage of infection in DCs. DCs were co-cultured with serially diluted ApoAct (Figure S2). We detected an increasing percentage of p24^+^ DCs with decreasing numbers of ApoAct.

We also performed kinetic experiments by first incubating DCs with HIV_BaL_ for 30 minutes, 1 hour, or 2 hours followed by addition of ApoAct. We observed the same reduction in p24 expression if the DCs were exposed to the virus for 30 min, 1 hour or 2 hours prior to addition of ApoAct (Figure S3A). Conversely, DCs were first exposed to ApoAct for 30 minutes, 1 hour, or 2 hours before addition of HIV_BaL_. Again, the percentages of p24^+^ DCs were similar for all three pre-incubation time points (Figure S3A). We also performed experiments with longer kinetics by adding ApoAct 16 hours after HIV_BaL_ infection of DCs or adding ApoAct 16 hours before infection. Again, reduced percentage of infected DCs was detected when they were co-cultured with ApoAct under any of these conditions (Figure S3B). ApoAct-mediated inhibition of HIV in DCs was also detected using three additional virus isolates (HIV_IIIB_ and primary isolates 207 and 208) (Figure S4).

### TNF-α released in conditioned media from DC/ApoAct partially blocks HIV infection in DCs

**Figure 4:**
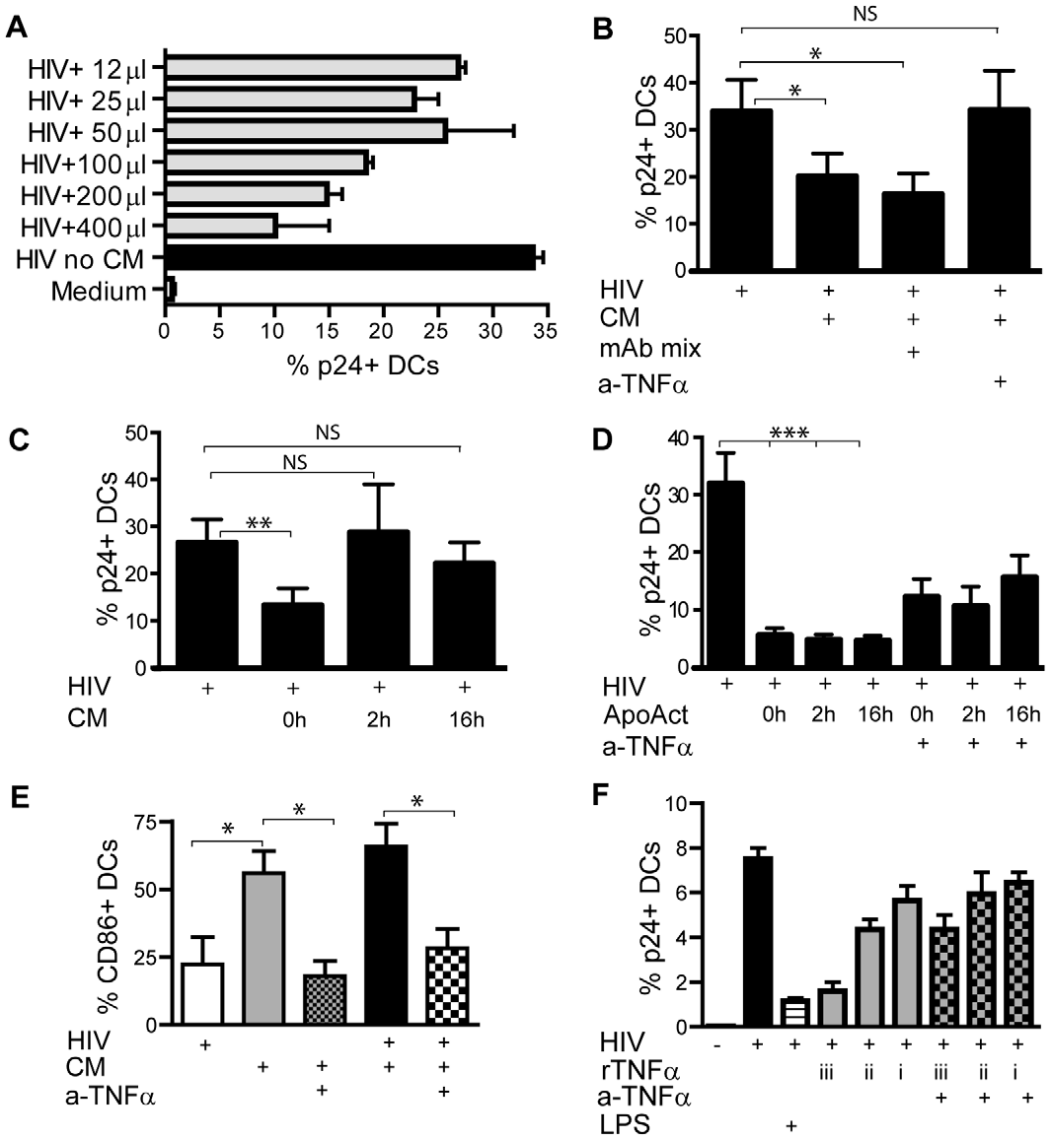
TNF-αreleased in co-cultures of DC and ApoAct mediate partial reduction in HIV infection and an increase in the percentage of CD86^**+**^ cells. (A) Conditioned medium (CM) was collected from co-cultures of DCs and ApoAct after 24 hours. Immature DCs were exposed to the supernatants in increasing concentrations (final volume, 1 ml) in the presence of HIV_BaL_. The percentage of intracellular p24^+^ cells was measured by flow cytometry after 7 days. One representative experiment out of two is shown. Two replicates were used to generate mean ± SD. No statistical analyses were performed for the CM dose-response analyses. (B) DCs were exposed to HIV_BaL_ (HIV); conditioned medium (CM) from DC and ApoAct co-cultures; or blocking mAbs to RANTES, MIP-1α, MIP-1β, and IFN-γmAb mixor to TNF-αa-TNF-α). The percentage of HIV-infected DCs was measured by flow cytometry in cells from eight donors. Significant differences were assessed by the non-parametric Wilcoxon matched-pairs test and are indicated by *p<0.05. (C) DCs were exposed to HIV, and CM was added at the same time as HIV (0 hours) or after 2 or 16 hours of incubation before assessment of intracellular p24 by flow cytometry after 7 days in five donors. Significant differences were assessed by the non-parametric Mann-Whitney *U*-test and are indicated as **p<0.01. (D) DCs were exposed to HIV and co-cultured with ApoAct added at the same time (0 hours) or after 2 or 16 hours after HIV infection in six donors. Significant differences were assessed by the non-parametric Mann-Whitney *U*-test and are indicated as ***p<0.001. The experiments were carried out in the presence or absence of blocking TNF-α mAbs in three donors and no statistical analyses were performed. Assessments of intracellular p24 by flow cytometry were performed 7 days after HIV exposure. (E) DCs were exposed to CM with or without simultaneous HIV exposure in the presence or absence of blocking TNF-α mAb, and the percentage of CD86^+^ cells was measured by flow cytometry after 7 days in five donors. Significant differences were assessed by the non-parametric Mann-Whitney *U*-test and are indicated as *p<0.05. (F) DCs were exposed to HIV in the presence of decreasing concentrations of rTNF-α (iii = 25 ng/ml, ii = 2500 pg/ml, i = 250 pg/ml) with or without simultaneous addition of TNF-α mAb. Assessments of intracellular p24 by flow cytometry were performed 7 days after HIV exposure. Data represent mean+SD from two donors obtained from one representative experiment out of three.

To assess whether conditioned medium (CM) collected from DC/ApoAct co-cultures reduces HIV infection, we collected supernatants after 24 hours of co-culture to use as CM. Different amounts of CM were added to cultures of immature DCs and HIV. There was a dose-dependent response in the reduction of p24 expression in DCs after exposure to CM (Figure 4A). To further elucidate the role of CM mediated reduction of HIV infection in DCs and to investigate possible candidate cytokines and/or chemokines for the effect, we added CM from DC/ApoAct cultures to DCs exposed to HIV_BaL_ (Figure 4B,C). A reduced p24 expression was detected in DCs exposed to HIV and CM as compared with DCs exposed to only HIV (*p<*0.05). The addition of blocking antibodies to MIP-1α, MIP-1β, IFN-γ and RANTES to the CM did not restore p24 expression, whereas addition of blocking antibody to TNF-α did (Figure 4B). CM added 2 hours or 16 hours after HIV exposure did not significantly reduce p24 expression in DCs (Figure 4C). However, ApoAct added 2 hours or 16 hours after HIV exposure reduced HIV p24 expression in DCs (Figure 4D; *p<*0.001). The HIV inhibitory effect by ApoAct was partially lost in the presence of TNF-α mAb (Figure 4D). In addition, the TNF-α mAb blocked CM-mediated up-regulation of CD86 in DCs (Figure 4E; *p<*0.05). We next treated immature DCs with different concentrations recombinant TNF-α and exposed them to HIV. There was a dose-dependent inhibition of HIV infection in DC by TNF-α where the highest concentration (25 ng/ml) showed an effective inhibition (Figure 4F). The HIV inhibitory effect by TNF-α was lost in the presence of TNF-α mAb (Figure 4F). These data show that TNF-α treatment can inhibit HIV infection in these DCs and that the HIV inhibitory effect by ApoAct can be partially explained by release of TNF-α.

### APOBEC3G-mediated HIV inhibition in DCs exposed to ApoAct

**Figure 5:**
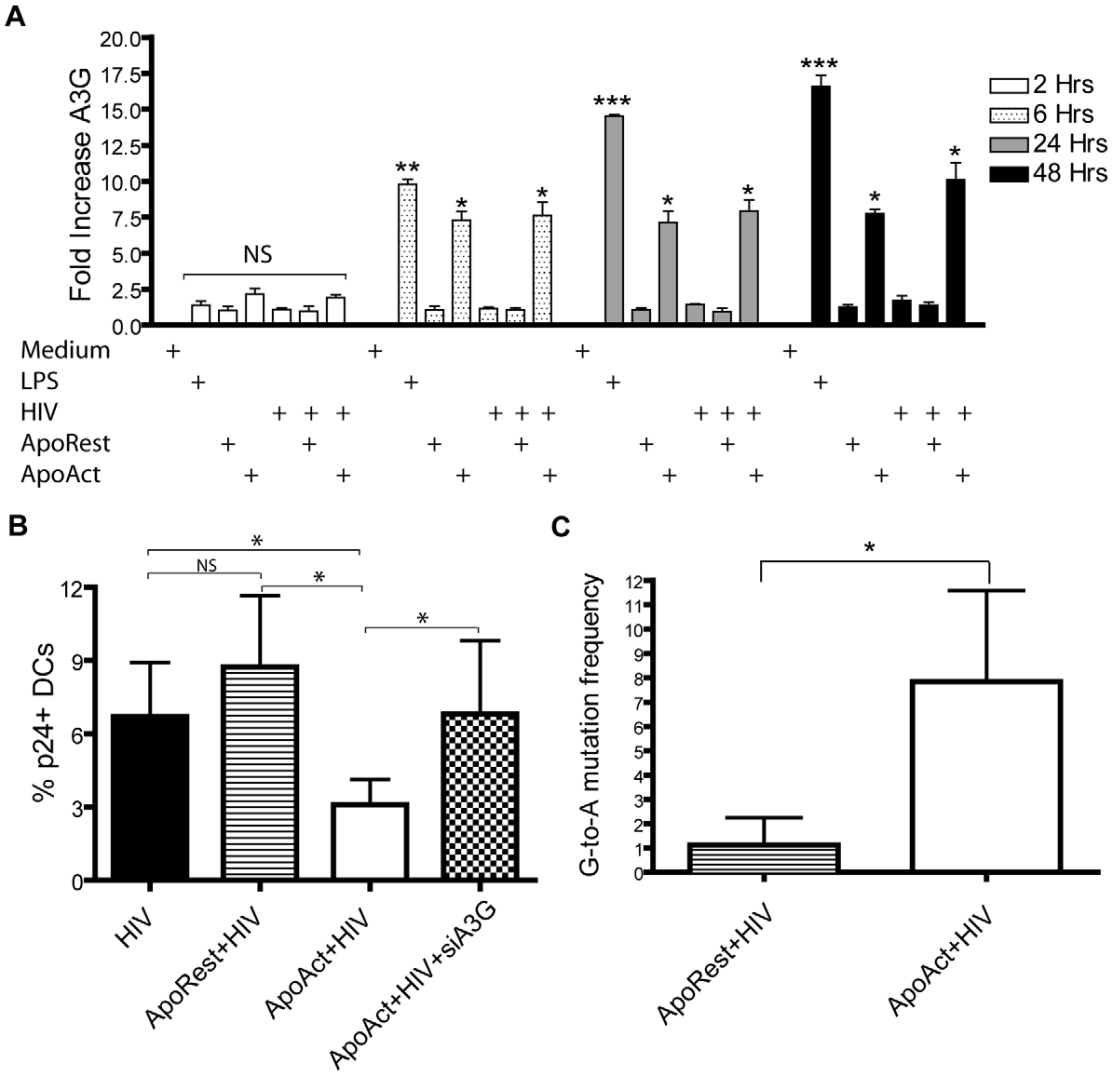
APOBEC3G mRNA expression and G-to-A hypermutations are induced after co-culturing with ApoAct but not ApoRest. (A) Real-time PCR was used to determine fold changes in mRNA for APOBEC3G in DCs co-cultured with LPS, resting apoptotic allogeneic CD4^+^ T cells (ApoRest), or activated apoptotic allogeneic CD4^+^ T cells (ApoAct). mRNA levels were compared with mRNA obtained from DCs cultured in medium at different time points (2 hours, 6 hours, 24 hours, and 48 hours). Data represent the mean ± SD from four different donors. Real-time PCR analyses of the 24 hours samples were repeated three times with similar values obtained. Significant differences were assessed by non-parametric Kruskal-Wallis test with Dunn's Multiple Comparison Test and are indicated by *p<0.05, **p<0.01, and ***p<0.001. Non-significant, NS. (B) Intracellular p24 expression was measured by flow cytometry in DC exposed to HIV, HIV and ApoRest, HIV and ApoAct non-targeted siRNA or in DC treated with APOBEC3G siRNA (siA3G) prior to exposure to HIV and ApoAct. Data represent mean±SD from six donors and analyses were performed seven days after infection. Significant differences were assessed by the non-parametric paired Wilcoxon test and are indicated as *p<0.05. (C) G-to-A mutations in HIV-1 *env* clones. HIV-1 *env* sequences were generated from HIV_BaL_ infected DCs co-cultured either with ApoRest or ApoAct. G-to-A mutation frequencies as compared with a HIV_BaL_ sequence obtained from DCs cultured with the same stock of HIV_BaL_ were measured in clones from HIV_BaL_ infected DCs co-cultured either with ApoRest (n = 35) or ApoAct (n = 26). Significant differences were assessed by the t-test (unpaired), and significance is indicated by *p<0.03.

The anti-viral factor APOBEC3G is induced upon DC maturation by LPS [25]. We therefore investigated whether APOBEC3G was induced after ApoAct-mediated DC maturation. RNA was prepared from DC co-cultures after 2, 6, 24, and 48 hours, and APOBEC3G expression was assessed by real-time PCR. We detected induction of APOBEC3G after LPS stimulation and after ApoAct exposure in DCs when analyzed at the time points 6, 24 and 48 hours. However, we did not detect APOBEC3G after 2 hours of incubation, which showed that the APOBEC3G detected was not derived from the ApoAct. DC co-cultures with ApoRest did not promote induction of APOBEC3G (Figure 5A). In control experiments, we used two different housekeeping genes; GAPDH and 18S RNA, to normalize the data and obtained a similar pattern of upregulation of APOBEC3G after stimulation with either LPS or ApoAct (Figure S5).

To investigate whether APOBEC3G induced by ApoAct played a role in the HIV inhibitory effect in DCs, we silenced APOBEC3G using siRNA. The transfection procedure per se, neither altered the expression levels of APOBEC3G, nor induced secretion of TNF-α (Figure S6). The efficiency of silencing as revealed by real-time PCR was in the range of 79–90% in four different DCs donors co-cultured with ApoAct. The silencing of APOBEC3G resulted in a significantly increased p24 frequency (p<0.03 Wilcoxon paired test) that returned to baseline infection levels (Figure 5B). These data show induction of APOBEC3G expression in DC upon co-culture with ApoAct but not ApoRest. Moreover, siRNA analyses revealed that the HIV inhibitory effect mediated by ApoAct can largely be explained by induction of APOBEC3G in DCs. To verify that APOBEC3 proteins exerted a function to restrict HIV infection in the DC, we sequenced a region in *env* and measured the frequencies of G-to-A mutations using the program HYPERMUT 2.0. We sequenced 6 clones from HIV_Ba-L_ infected DC of which one was used as reference sequence, 35 clones from HIV_Ba-L_ infected DC co-cultured with ApoRest and 26 from DC co-cultured with ApoAct (Table 1). In total 21,000 base pairs were sequenced from ApoRest co-cultures and 15,600 from ApoAct. None of the clones sequenced from ApoRest co-cultures were found to have significant induction of G-to-A hypermutations according to the HYPERMUT program, while 3 clones had significant p<0.05 Fisher exact *p*-value in co-cultures with ApoAct. The total number of G-to-A mutations detected in ApoRest co-cultures were 39 while 201 mutations were detected in clones derived from ApoAct co-cultures. The average numbers of G-to-A mutations per 100 base pairs were 0.09 and 1.288 in the ApoRest and ApoAct co-cultures, respectively. G-to-A mutations were detected both in the GG and GA context. There was a significant difference (*p*<0.03 unpaired t-test) in the mutation frequency in clones obtained from ApoRest DC co-cultures compared with ApoAct (Figure 5C). These data demonstrate signs of APOBEC3G activity in DCs after co-culture with ApoAct.

**Table 1:** Summary of G-to-A mutation frequencies in HIV-1 *env* sequences obtained from HIV_BaL_ infected DC co-cultures.

Item Analyzed	DC co-cultured with ApoRest	DC co-cultured with ApoAct
Total no. of clonessequenced	35	26
Total no. of base pairssequenced	21,000	15,600
Total no. of clones withFisher Exact *p*-value <0.05	0	3
Total no. of G-to-Amutations	39	201
No. of G-to-A mutations(GG context)	8	60
No. of G-to-A mutations(GA context)	11	40
Avg no. of G-to-Amutations/100 bp	0.090	1.288

## Discussion

Infection of DCs is likely to be an important contributor to the spread of HIV by capturing virus at sites of viral entry followed by migration to lymphoid tissues, which constitute an optimal site for HIV transmission to T cells [30]. Production of HIV can be blocked by exposure of DCs to maturation stimuli such as for example LPS [3], [10], [48]. Here we have demonstrated that apoptotic activated CD4*^+^* T cells (ApoAct), either uninfected or recently HIV-infected (ApoInf), induce expression of co-stimulatory molecules, release of cytokines as well as reduce HIV infection in DCs, whereas unactivated apoptotic CD4^+^ T cell (ApoRest) do not. We show that induction of the restriction factor APOBEC3G was important for the antiviral activity in the DCs. However, these findings do not preclude involvement of other restriction factors such as for example APOBEC3F [25]. These findings have implications for viral transmission as DCs are likely to be one of the first target cells upon transmission when encountering infected cells and/or free virus.

We found significantly reduced HIV infection in DCs that had been co-cultured with ApoAct but not in DCs co-cultured with ApoRest. It was previously suggested that alloantigen-induced immune responses may reduce HIV susceptibility in CD4^+^ T cells [47], [49]. In the present study, we show that both allogeneic and autologous ApoAct are able to provide DCs with a maturation signal; however, a prerequisite for the induction of DC maturation and anti-viral activity was that both the allogeneic and autologous cells were activated before induction of apoptosis and subsequent co-culturing with DCs. There was no difference in HIV-infected or uninfected ApoAct to induce DC maturation or cytokine release and that there was no clear induction of DC maturation by the virus itself-rather it was the activated apoptotic cell component that made a difference. It should be noted that we removed cell debris from the virus preparations to avoid possible contaminating dead cells from the activated PBMCs used for viral growth.

We also show that part of the anti-HIV activity is mediated by TNF-α released into the supernatant after co-culture with ApoAct. TNF-α is an example of a cytokine that can differentially inhibit or promote HIV replication depending on the target cell type, which adds to the complexity of events occurring *in vivo* [14]. We detected up-regulation of APOBEC3G mRNA expression in the DC co-cultures with ApoAct indicating that inhibition might occur at least in part at the transcriptional level [25], [50]. Furthermore, PCR analyses showed that the amount of HIV-DNA present in DCs after co-culture with ApoAct is lower as compared to DCs co-cultured with ApoRest, which is in concordance with these findings, although it should be pointed out that there was not a complete elimination of HIV p24^+^ cells in the cultures. In addition, our data suggests that the inhibition was occurring both pre- and post-integration. We showed that the anti-retroviral restriction factor APOBEC3G was important for the anti-viral effect in the present system by silencing APOBEC3G in DCs prior to addition of HIV and ApoAct. Signs of APOBEC3 activity were measured by sequence analyses of a region in *env*, which revealed significant induction of G-to-A hypermutations in co-cultures with ApoAct but not ApoRest. Of note is that the mutations occurred both in a GG and GA context, which implies that the mutations were induced at least in part by APOBEC3G as GG dinucleotides are target sites specific for APOBEC3G [23]. APOBEC3A was also reported to induce G-to-A mutations in a GG context but there is currently little data showing a HIV restricting role for APOBEC3A [23]. Several family members of the APOBEC3 family were reported to induce G-to-A hypermutations leading to HIV restriction in a GA context of which APOBEC3F is well studied [23]. The present system used a complete HIV_BaL_ with capacity to produce Vif, a protein know to counteract the antiviral effect by APOBEC3G [23]. We observed the antiviral activity after a 7-10 fold increase in APOBEC3G mRNA expression, which appeared sufficient to restrict HIV-1 replication in the DCs.

We previously reported that apoptotic PBMCs obtained after 24 hours of activation with either CD3/CD28 mAbs or PHA efficiently support DC maturation, while 4 days of PHA activation prior to apoptosis induction resulted in a reduced capacity (although still readily detected) to induce CD86 expression [29]. We therefore here activated CD4^+^ T cells with CD3/CD28 mAbs for 24 hours and induced apoptosis no more than 3–4 days after initial T cell activation in either uninfected or HIV infected CD4^+^ T cells. Hence, we induced apoptosis before extensive HIV-mediated apoptosis occurred in the cultures. Longer exposures to activation or activation induced cell death as well as resting apoptotic cells do not promote DC maturation and such cells are instead involved in peripheral tolerance mechanisms [29], [51]–[53].

Cell death is a hallmark of HIV pathogenesis [31]. SIV infection is accompanied by an early depletion of CD4^+^ T cells in the gut of macaques, and progression to AIDS is characterized by depletion of CD4^+^ T cells [54]. However, it is not only HIV-infected cells that die during HIV infection. There is also a massive depletion of non-infected bystander cells. HIV-mediated bystander killing was suggested to occur through viral molecules, such as Nef, Tat, Vpr, Vpu, Vif, and Env [55]. Increased apoptotic cell death occurs during HIV infection, but the question of which type of cell death that is induced in HIV-infected cells *in vivo* is still somewhat controversial [31], [56]. It is largely unknown whether the HIV-infected cells that undergo cell death *in vivo* are dying an immunogenic cell death. Nevertheless, the contribution of different apoptotic cells during HIV infection *in vivo* is likely to be complex and the integrated net effect may in part depend on whether the apoptotic cells are dying in a state when they are able to induce cytokine production and DC maturation. Hence, we propose to make a clear distinction between recently activated cells, cells that have been activated for a prolonged period before apoptosis, and resting apoptotic T cells regarding subsequent DC-induced responses. The data presented here suggest that apoptotic CD4^+^ T cells that were efficiently activated prior to their cell death may be an important mechanism to reduce HIV infection of DCs. Aberrant immune activation is another characteristic hall mark of HIV infection [57] and it remains to be investigated whether the apoptotic lymphocytes formed during HIV infection has capacity to reduce HIV infection in DCs.

Together these findings show that apoptotic activated CD4^+^ T cells, either uninfected or HIV-infected, have the capacity to induce DC maturation and cytokine release of TNF-α, MIP-1α, MIP-1β, and MCP-1. In addition, we detected a reduced percentage of HIV-infected DCs after exposure to ApoAct or conditioned media from DC/ApoAct co-cultures but not with ApoRest. We revealed that TNF-α was at least partially mediating the maturation and reduced HIV infection in DCs. We could show that the anti-viral effect induced by ApoAct was largely dependent on upregulation of the restriction factor APOBEC3G in the DCs leading to induction of G-to-A hypermutations. These findings have implications for transmission of HIV and HIV pathogenesis involving interactions between HIV, apoptotic cells and DCs.

## Supporting Information

Figure S1 **Increase in the percentage of CD86^+^ cells and reduced percentage of HIV-infected DCs after exposure to either autologous or allogeneic ApoAct.** (A) The capacity to induce CD86 expression in DCs by either autologous (AutoApoAct) or allogeneic (AlloApoAct) apoptotic anti-CD3– and anti-CD28–activated CD4^+^ T cells was measured by flow cytometry after 72 hours in co-culture. The mean ± SD from nine donors is shown. (B) The capacities of AutoApoAct and AlloApoAct in reducing HIV infection in DCs were compared. Results are depicted as the mean ± SD from seven donors. Significant differences were assessed by the non-parametric Mann-Whitney *U*-test and are indicated by *p<0.05. Non-significant differences, NS.
(EPS)

Figure S2 **Dose-response effect of ApoAct on the percentage of HIV-infected DCs.** A dose-response assessment was performed by adding decreasing amounts of ApoAct (allogeneic) starting with a 1∶1 DC:ApoAct ratio. The number of DCs was kept constant, and the ApoAct were serially diluted (*n* = 4). HIV p24 expression in DCs was assessed by flow cytometry after 7 days. Data represent the mean ± SD from four different donors.
(EPS)

Figure S3 **Reduced percentage of HIV infection in DCs co-cultured with ApoAct both pre- and post-HIVBaL exposure.** Immature DCs were exposed to HIV_BaL_ (HIV) or both HIV and apoptotic anti-CD3– and anti-CD28–activated allogeneic CD4^+^ T cells (ApoAct). (A) ApoAct were added at the same time as the virus (ApoAct+HIV) or at 30 minutes, 1 hour, or 2 hours prior to addition of HIV. Conversely, in some cultures the DCs were first incubated with HIV for 30 minutes, 1 hour, or 2 hours prior to addition of ApoAct. The percentage of infected DCs was assessed by flow cytometry of intracellular p24 staining after 7 days. The resulting mean ± SD from four donors is shown. (B) Longer kinetic experiments were also performed by incubating DCs with HIV for 16 hours before adding the ApoAct, or by incubating the DCs for 16 hours with ApoAct before adding the virus. The mean ± SD from four donors is shown. No statistical analyses were performed.
(EPS)

Figure S4 **ApoAct inhibit infection of different HIV isolates in DCs.** Immature DCs were exposed to HIV_BaL_; HIV_IIIB_; or two different primary isolates, 207 and 208, in the presence (white bars) or absence (black bars) of ApoAct (allogeneic). The percentage of infected DCs was assessed by flow cytometry of intracellular p24 staining after 7 days. Data represent the mean ± SD from two different donors.
(EPS)

Figure S5 **APOBEC3G mRNA expression are induced after co-culturing with either LPS or ApoAct after normalization to two different reference genes.** Real-time PCR was used to determine fold changes in mRNA for APOBEC3G in DCs co-cultured with LPS, resting apoptotic allogeneic CD4^+^ T cells (ApoRest), or activated apoptotic allogeneic CD4^+^ T cells (ApoAct). Cycle threshold values for APOBEC3G were normalized either to the value for GAPDH or 18s RNA. mRNA levels were compared with DCs cultured in medium for 24 hours. Data represent the mean ± SD from four different donors.
(EPS)

Figure S6 **Efficiency of APOBEC3G silencing in DCs by siRNA.** (A) To silence APOBEC3G, the DCs were either transfected with APOBEC3G specific siRNA (siA3G), Non-Targeting siRNA (siNonTarget) or not exposed to the transfection procedure. Real-time PCR was used to determine the efficiency of the silencing by measuring the fold changes in mRNA for APOBEC3G in DCs co-cultured with LPS, resting apoptotic allogeneic CD4^+^ T cells (ApoRest), or activated apoptotic allogeneic CD4^+^ T cells (ApoAct). The mRNA levels were compared with DCs cultured in medium for 24 hours. Results are representative of two donors. (B) Supernatant from DCs co-cultured with LPS, ApoRest, or ApoAct that were either transfected with siA3G, siNonTarget or not exposed to the transfection procedure, were collected after 24 hours and analyzed for the presence of TNF-α by performing an ELISA. Results are representative of two donors.
(EPS)
